# Evaluation of Tibial Fixation Devices for Quadrupled Hamstring ACL Reconstruction

**DOI:** 10.1177/23259671221096107

**Published:** 2022-05-11

**Authors:** Elias Ammann, Andreas Hecker, Elias Bachmann, Jess G. Snedeker, Sandro F. Fucentese

**Affiliations:** † Balgrist University Hospital, Zürich, Switzerland.; *Investigation performed at Balgrist University Hospital, Zürich, Switzerland*

**Keywords:** anterior cruciate ligament, tibia, fixation, biomechanics

## Abstract

**Background:**

Shortcomings to tibial-side fixation have been reported as causes of failure after anterior cruciate ligament reconstruction. Adjustable-loop suspensory devices have become popular; however, no comparison with hybrid fixation (ie, interference screw and cortical button) exists to our knowledge.

**Purpose:**

The purpose of this study was to compare the biomechanical properties of adjustable loop devices (ALDs) in full-tunnel and closed-socket configurations in relation to hybrid fixation. We hypothesized that primary stability of fixation by a tibial ALD will not be inferior to hybrid fixation.

**Study Design:**

Controlled laboratory study.

**Methods:**

Tibial fixation of a quadrupled tendon graft was biomechanically investigated in a porcine tibia–bovine tendon model using 5 techniques (n = 6 specimens each). The tested constructs included hybrid fixation with a cortical fixation button and interference screw (group 1), single cortical fixation with the full-tunnel technique using an open-suture strand button (group 2) or an ALD (group 3), or closed-socket fixation using 2 different types of ALDs (groups 4 and 5). Each specimen was evaluated using a materials testing machine (1000 cycles from 50-250 N and pull to failure). Force at failure, cyclic displacement, stiffness, and ability to pretension the graft during insertion were compared among the groups.

**Results:**

No differences in ultimate load to failure were found between the ALD constructs (groups 3, 4, and 5) and hybrid fixation (group 1). Cyclic displacement was significantly higher in group 2 vs all other groups (*P* < .001); however, no difference was observed in groups 3, 4, and 5 as compared with group 1. The remaining tension on the construct after fixation was significantly higher in groups 3 and 4 vs groups 1, 2, and 5 (*P* < .02 for all comparisons), irrespective of whether a full-tunnel or closed-socket approach was used.

**Conclusion:**

Tibial anterior cruciate ligament graft fixation with knotless ALDs achieved comparable results with hybrid fixation in the full-tunnel and closed-socket techniques. The retention of graft tension appears to be biomechanically more relevant than tunnel type.

**Clinical Relevance:**

The study findings emphasize the importance of the tension at which fixation is performed.

In anterior cruciate ligament (ACL) reconstruction, a 4-strand hamstring autograft is a commonly used graft option.^4,30,41^ A quadrupled semitendinosus graft provides a sufficient length in most cases and, in general, is superior in diameter as compared with a 4-strand semitendinosus-gracilis graft. One reason for short- and long-term failure is the fixation method, particularly on the tibial side.^5,7,18,22^ Despite the superiority of hybrid fixation with combined intratunnel (ie, aperture interference screw) and extratunnel (ie, cortical suspensory) devices,^5,21,25^ single interference screw fixation at the tibia is a common practice.^20,45^

With a closed-socket technique (popularized as the “all-inside” technique), the use of an interference screw is not practiced. Several potential advantages of closed-socket ACL reconstruction have been described, such as bone preservation attributed to the drilling of closed-socket tunnels, lower postoperative pain scores, and the ability to retighten the construct using adjustable loop suspensory fixation devices.^12,24,26,27,36^ Nevertheless, many surgeons hesitate to adopt the closed-socket technique with concerns of the lacking aperture fixation, which has been suspected to cause elongation and delayed graft-to-bone healing.^6,45^ Although some studies have revealed superior graft incorporation related to the closed-socket technique,^11,43^ others have shown tunnel widening and assumed a “bungee” or “windshield wiper” effect to be responsible for increased graft motion and reduced graft-to-bone healing.^16,29,40^

While it appears from a biomechanical point of view that suspensory closed-socket devices are not inferior to single interference screw fixation,^8,31,42,46^ there has been no study comparing these constructs, to our knowledge. In the current study, we used a porcine tibia–bovine tendon model to compare adjustable loop devices (ALDs) with hybrid full-tunnel fixation at the tibia. We hypothesized that the biomechanical properties of tibial fixation with ALDs (total load to failure, cyclic elongation, and stiffness) will not differ significantly from hybrid fixation, irrespective of conducting the full-tunnel or closed-socket technique. In a secondary analysis, we assessed the relationship of construct tension before testing to cyclic displacement after preconditioning.

## Methods

**Figure 1.**
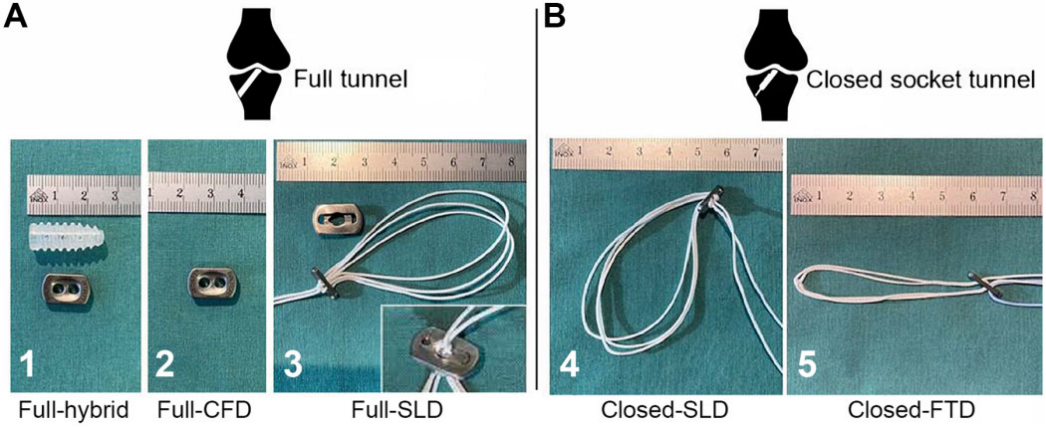
The tibial fixation devices that were tested by construct group. (A) Full-tunnel technique with hybrid fixation (1), cortical suspensory fixation for open-suture strand (2), and ALD based on a modified sling lock mechanism and extension button (3). (B) Closed-socket configuration with ALD based on a modified sling lock mechanism (4) and ALD based on a finger trap mechanism (5). ALD, adjustable loop device; CFD, cortical fixation device; FTD, finger trap device; SLD, sling lock device.

Five fixation methods were investigated in a commonly used porcine tibia–bovine tendon in vitro model (Figure 1).^1,15,17,23,33^ A total of 30 fresh-frozen porcine knees and bovine extensor digitorum tendons were obtained from the local butcher. Six grafts were tested for each fixation method. All procedures were performed by 2 orthopaedic senior registrars (E.A. and A.H.) of the hospital’s knee team.

The full-tunnel technique was used in groups 1, 2, and 3, testing 3 fixation methods. Group 1 consisted of a hybrid fixation using a 9-mm bioabsorbable poly(L-lactide-co-D, L-lactide) interference screw (Megafix, Karl Storz SE & Co KG) and a cortical fixation device (CFD; Endotack, Karl Storz). An isolated single cortical suspensory fixation (Endotack) was tested in group 2. In group 3, an ALD based on a modified sling lock mechanism (VariLoop tibial, ZuriMED Technologies AG) was used. As a larger surface is necessary to cover the naturally larger bone tunnel entrance in the full-tunnel technique, a custom-made extension button was machined for this study (Figure 1).

In groups 4 and 5, a closed-socket tunnel configuration was applied (ie, closed-socket technique). Fixation in group 4 was performed with the same ALD as in group 3 without using the additional extension button. In group 5, an ALD based on the finger trap mechanism was tested (TightRope-RT, Arthrex).

### Specimen Preparation

After thaw and removal of the femur and all soft tissue from the tibias, the bone tunnels were drilled. For groups 1, 2, and 3, full tunnels (diameter, 8 mm; length, 50 mm) were drilled from the anteromedial tibial surface to the anatomic porcine ACL footprint using a target guide, a guide wire, and a cannulated 8-mm bone drill. To create closed-socket tunnels for groups 4 and 5, a 30-mm socket with a diameter of 8 mm was drilled over the guide wire using a retrograde drill starting from the ACL footprint. The remaining 10 mm was completed with a cannulated 3.5-mm bone drill. All tendons were prepared on a graft preparation board and sized to a length of 240 mm, and tendon ends were baseball-style whipstitched in both directions over the last 2 cm with No. 2 FiberWire sutures (Arthrex).

**Figure 2.**
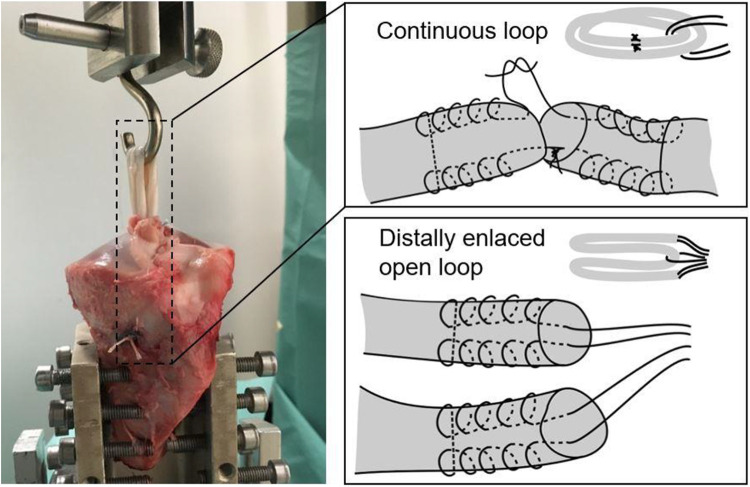
(Left) Mounting of the tibia graft construct on a universal materials testing machine. The proximal end of the graft is secured to a steel hook and aligned parallel to the machine loading axis. (Right) Schematic representations of the quadrupled graft preparation techniques with either continuous loop with an adjustable loop device (groups 3, 4, and 5) or open ends (groups 1 and 2).

The tendon grafts of group 1 and 2 were quadrupled and distally enlaced with a No. 6 Ethibond loop (Ethicon Inc). For the ALD in groups 3 to 5, sutures of both graft ends were knotted with 7 surgeon’s knots and manually tightened to achieve continuous loop grafts that were quadrupled (Figure 2). This allowed enlacing the grafts in the suture loops of the ALDs. This method for ALD fixation was chosen since it appears to be the most practicable technique that achieves results comparable with other, more laborious techniques.^49^

### Graft Insertion and Fixation

Before graft insertion, tibias were mounted on a universal materials testing machine (Zwick 1456, ZwickRoell GmbH & Co KG) (Figure 2). Each tibia was rigidly fixed in a 45° angulated, 60-mm aluminum cylinder to achieve tension concentric to the bone tunnel, simulating a worst-case load condition. The *x-y* table with the mounted tibias allowed unconstrained travel in the *x* and *y* directions. The femoral side was represented by a steel hook over which the quadrupled graft was folded. The steel hook was connected to a load cell and the uniaxial drive of the tension machine.

Before insertion, the diameter of the quadrupled graft was measured with a graft-sizing block resulting in an either 8- or 9-mm graft. Bone tunnels and sockets were augmented with a 9-mm dilator for 9-mm grafts. Quadrupled grafts of the full tunnel in groups 1 to 3 were pulled from distal to proximal through the bone tunnel with a passing suture and set in the hook proximally. Intratunnel fixation of group 1 was achieved with a 9-mm–diameter, 23-mm–length interference screw (Megafix, Karl Storz) that was inserted over a guide wire while as much tension as manually possible was applied on the distal suture ends. Cortical fixation of groups 1 and 2 was performed by passing the sutures through an Endotack suspensory button and knotting the sutures 7 times. In group 3, the graft loop was also pulled through the bone tunnel from distal to proximal and fixed cortically on the tibia using the sling lock device and the custom-made extension button. Manual tension was applied to tighten the sling lock mechanism before cutting the sutures 10 mm distal of the blocking mechanism.

The grafts in groups 4 and 5 were inserted from the articular side through the bone tunnel with a passing suture until the grafts reached a depth of 25 mm in the bone socket. The implant-graft constructs were then tensioned with the adjustable loop systems. While suture ends of group 4 were cut 10 mm distal from the modified sling lock mechanism, 5 surgeons’ knots were applied on the suture ends of group 5, as recent studies reported better mechanical properties after knot tying with a button based on the finger trap mechanism.^10,34^ In each group, graft fixation was performed with the maximal tension that was manually achievable with the fixation mechanism.

### Biomechanical Testing

Biomechanical testing was performed in 3 phases. In the first phase, the constructs were preconditioned with 10 cycles between 10 and 50 N, representing a phase of low stress to the construct. In the second phase, cyclic testing from 50 to 250 N was initiated (1000 cycles, force controlled at 2 mm/s). An ultimate pull-to-failure test was conducted at a speed of 20 mm/min in the third phase.^32,37^

Maximum tensioning forces (in newtons) on the graft were recorded during and after insertion, and the tension for each construct was assessed after cortical fixation and before testing. The displacement after preconditioning was measured, representing the initial laxity just after surgery. Cyclic displacement (in millimeters), stiffness (in newtons per millimeter), and ultimate failure force (in newtons) were determined per the recorded load-displacement curve.

### Statistical Analysis

For cyclic tests without complete unloading, a sample size of 6 per group was adapted from previous literature.^2,9,34,38^ A priori power calculations were performed for cyclic displacement based on pilot and literature data.^38^ Effect sizes for this experiment were anticipated to be large (Cohen *d* ≥ 3); therefore, 5 samples per group seemed to be adequate to yield sufficient statistical power (*P* 0.98).

Standard software (Prism Version 7.03, GraphPad) was used for statistical analysis. Kolmogorov-Smirnov testing was used to test whether the values were adequately fit by Gaussian distributions. Retained tension, stiffness, cyclic displacement, and ultimate tensile strength were compared among fixation devices using 1-way analysis of variance, and a Tukey multiple-comparison test was used for post hoc testing (α = .05). Data that were nonnormally distributed were compared using Kruskal-Wallis analysis of variance.

## Results

Table 1 shows the failure modes that were visually identified after pullout testing. The most prevalent mode of failure in constructs with the full-tunnel technique was rupture of sutures, whereas constructs with the closed-socket technique most often failed at the button–bone junction.

**Table 1:** Failure Modes of the Different Constructs After Pullout Testing*^a^*

	Failure Mode, n			
Group: Construct*^b^*	Graft-Suture Interface Failure	Graft Rupture	Fixation Suture Rupture	Graft and Button Pulled Through Bone Tunnel
1: Full hybrid	1	0	4	1
2: Full CFD	1	0	5	0
3: Full SLD	0	1	5	0
4: Closed SLD	0	0	1	5
5: Closed FTD	2	1	0	3

*^a^* CFD, cortical fixation device; FTD, finger trap device; SLD, sling lock device.

*^b^* Each group, n = 6.

**Figure 3.**
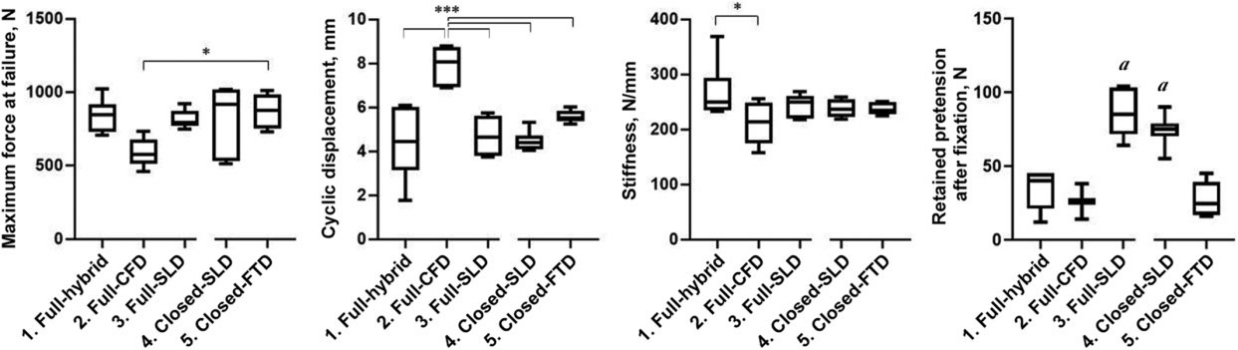
Box plots show the results of biomechanical testing for the 5 study constructs. The middle line represents the median; the box, interquartile range; and the whiskers, the minimum and maximum values. Significant difference between groups: *P < .05. ***P < .001. *^a^* Significant difference vs groups 1, 2, and 5 (*P* < .05). CFD, cortical fixation device; FTD, finger trap device; SLD, sling lock device.

Figure 3 shows the results of the biomechanical testing by construct group. Ultimate failure force differed significantly between group 2 (full CFD) and group 5 (closed finger trap device), showing superiority of the latter (*P* = .013). Regarding cyclic displacement, similar values were found in all groups except in group 2 (full CFD), in which the displacement was significantly higher than all other groups (*P* < .001). In accordance, stiffness was lowest in group 2 (full CFD) and differed significantly from group 1 (full hybrid; *P* = .033); this was the only significant difference in stiffness.

**Figure 4.**
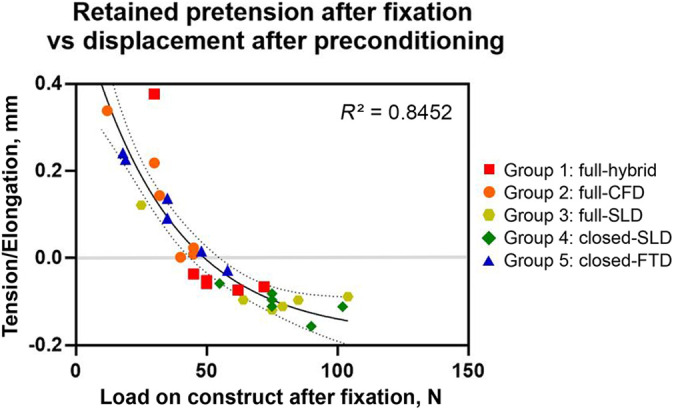
Correlation of retained pretension and initial displacement of different devices. Values less than 0 (gray line) indicate that the implanted graft was still under tension after preconditioning (eg, tendon was still stretched by 0.1 mm), and values greater than 0 indicate that the construct experienced elongation. Nonlinear regression curve (*R* ^2^ = 0.85) was assessed with goodness of fit (sum of squares) and positively tested for homoscedasticity. The dotted lines indicate 95% CIs. CFD, cortical fixation device; FTD, finger trap device; SLD, sling lock device.

The remaining tension on the construct after graft fixation was significantly higher when fixation was performed with ALDs using the modified sling lock mechanism (groups 3 and 4) as compared with groups 1, 2, and 5 (*P* < .02 for all comparisons) (Figure 3, Table 2); this was independent of whether a full-tunnel or closed-socket approach was used. A comparison of construct tension before testing and cyclic displacement after preconditioning across all constructs showed a clear nonlinear relationship (*R* ^2^ = 0.85) between those factors (Figure 4).

**Table 2:** Tension on the Graft After Fixation*^a^*

Group: Construct*^b^*	Tension, N
1: Full hybrid	26.0 ± 12.0
2: Full CFD	34.4 ± 13.9
3: Full SLD	86.8 ± 16.7^c^
4: Closed SLD	74.2 ± 11.1^c^
5: Closed FTD	27.3 ± 11.5

*^a^* Data are reported as mean ± SD. CFD, cortical fixation device; FTD, finger trap device; SLD, sling lock device.

*^b^* Each group, n = 6.

*^c^* A significantly higher tension was found in groups 3 and 4 vs groups 1, 2, and 5 (*P* < .05).

## Discussion

The most important findings of our study were first that tibial ACL graft fixation using a knotless ALD (groups 3-5) was mechanically comparable with hybrid fixation (group 1) regarding total load to failure, stiffness, and cyclic elongation. Furthermore, ALDs (groups 3-5) provided comparable fixation properties with hybrid fixation (group 1) irrespective of a full-tunnel or closed-socket technique. Conversely, solitary cortical fixation with an open-suture strand button (group 2) was inferior. Finally, analysis of performance after cyclical loading showed that retained graft pretension depended strongly on the device and related technique, favoring devices that limit slippage during loading.

Preexisting literature has demonstrated superiority of tibial hybrid fixation as compared with single interference screw and single cortical suspensory fixation.^5,21,25^ In the present study, no difference was found with hybrid fixation (group 1) when single extracortical fixation was performed with an ALD (groups 3-5). Moreover, it appears that tibial fixation with ALDs can compete with hybrid fixation independent of the performed technique, since a significant difference did not occur in the full-tunnel technique (group 3) or the closed-socket technique (group 4 and 5) when these ALDs were applied.

A solitary cortical suspensory fixation with an open-suture strand button in the full-tunnel technique (group 2) seems to be insufficient, showing significantly inferior fixation properties as compared with the tested ALDs (groups 3-5) and the hybrid fixation (group 1). This finding stands in accordance with former studies that have reported better fixation properties with hybrid fixation in a full-tunnel technique.^5,13,21^ However, this inferiority of single cortical suspensory fixation does not apply to the tested ALDs (groups 3-5).

In the present study, an ALD based on a modified sling lock mechanism was tested in a full-tunnel technique (group 3).^19^ The fixation mechanism is based on a double-pulley system allowing the surgeon to pretension the graft with 4 times the manual pulling force. Here a smaller button designed for a closed-socket fixation was adapted within a larger housing to accommodate use in a full-tunnel technique. This housing was sized to accord with the CFD (Endotack button) tested in group 2 and is not available on the market; however, the device is comparable with other commercially available products that help enlarge the surface of ALDs in a suspensory full-tunnel technique. Cyclic displacement with this double-pulley ALD in the full-tunnel technique (group 3) was significantly lower than the open-suture strand button (group 2) and did not differ from hybrid fixation (group 1) or the ALDs with the closed-socket technique (groups 4 and 5).

Regarding final failure of fixation constructs, it appeared that fixation with the closed-socket technique is prone to bone–button interface failure, whereas the most common mode of failure with the full-tunnel technique was suture rupture. In the closed-socket technique, small buttons can be applied, which might be beneficial for slim patients. However, the smaller buttons come with higher pressure on the cortical surface and should be used carefully when bone quality is suspect. In the full-tunnel technique, stronger suture material could increase the maximum load at failure, yet the mean maximum load at failure in a worst-case scenario was >800 N in all but group 2 (full CFD). As such, ultimate failure of tibial fixation seems to be a minor concern vs cyclic displacement that occurs owing to repetitive stress.

On the premise that fixation quality is reflected as retained graft pretension, we observed a clear nonlinear relationship between retained tension and creep displacement upon cyclical loading based on analysis across all tested constructs. The nonlinear relationship can be explained by the “stretching out” or “uncrimping” of crimped tendon fibrils, which occurs from mechanically loading the tendon up to 2% strain. This region is responsible for a nonlinear stress/strain curve because the slope of the toe region is not linear.^44^ Improved ability to retain tension through use of an ALD is generally consistent with the stronger fixation of these devices as compared with the open-suture strand button. Our data suggest that performance differences related to a surgeon’s choice between the closed-socket and full-tunnel techniques diminish when one uses full-tunnel devices that effectively retain graft tension. Moreover, the characteristic of tensioning is a general advantage of ALDs that is not reserved for tibial fixation, and surgeons preferring a tibial interference screw may consider using an ALD for femoral fixation to have the opportunity to retension the graft from proximal after tibial fixation.

It has already been shown that reconstruction stiffness is dependent on the initially applied graft tension^14^; however, less is known regarding how much pretension on a hamstring graft should be optimally applied for ACL reconstruction.^3,47,48^ The findings of the present study elucidate the relationship between fixation slippage during cyclic loading and the expected degree of tension loss. This is in alignment with Yasuda et al,^47^ who conducted a prospective clinical study with 70 patients and concluded that the initial relatively high tension in the graft (80 N) decreases postoperative looseness in the knee. Other research groups recommend an optimal graft pretension of 90 N.^39^ Noyes et al^35^ strongly suggested the need for increased load and graft cycling during implantation to remove residual elongation after ACL reconstruction.

Despite the expected benefits of adequately retaining graft tension, there are concerns about overtightening an ACL reconstruction and how this may result in joint stiffness and/or elevated tibiofemoral cartilage stress.^28^ Nevertheless, weak tibial fixation is frequently cited as a potential cause of graft failure,^5,7,18,22^ and the development of tibial-side fixation devices remains an active field. Our data suggest that regardless of the applied technique, retained fixation is reflective on minimizing residual elongation. We speculate that it may play a role in clinical outcome, and this should be investigated in further clinical studies.

### Limitations

Of course, there are several limitations to consider when judging the relevance of these findings. First, the study was designed as a biomechanical in vitro model using porcine tibias and bovine tendons. The findings cannot be applied in a clinical setting without caution; yet, these specimens are commonly used for biomechanical ACL testing, as their biomechanical properties are similar to human bone and tendon, respectively.^1,17,23,33^ Furthermore, this is a time-zero biomechanical study and does not explore any postoperative issues, such as graft incorporation or tunnel widening. In addition, the tested constructs were a combination of bone, fixation device, sutures, and tendon. We did not differentiate how local displacements cumulated to the overall mechanical response. However, graft preparation and suturing were standardized using 2 techniques: a continuous loop technique for fixation with an ALD (groups 3-5) and a quadrupling technique for fixation with the knotting button (groups 1 and 2). While clinical studies will be required to validate the clinical relevance of the experimental conclusions that we draw, these data do provide interesting insight and add unique perspective to the wide body of existing experimental literature.

## Conclusion

Tibial fixation of quadrupled ACL grafts with knotless ALDs achieves comparable results with hybrid fixation in the full-tunnel and closed-socket techniques. The findings of the present study emphasize the importance of the tension at which fixation is performed.
